# Effects of Internet of Things-based power cycling and neuromuscular training on pain and walking ability in elderly patients with KOA: protocol for a randomized controlled trial

**DOI:** 10.1186/s13063-022-06946-x

**Published:** 2022-12-13

**Authors:** Yujia Zhang, Suhang Xie, Xiaoyi Wang, Kangping Song, Lin Wang, Ruishi Zhang, Yuan Feng, Chengqi He

**Affiliations:** 1 Rehabilitation Medicine Center, West China Hospital, Sichuan University, Chengdu, 610041 Sichuan People’s Republic of China; 2 Rehabilitation Key Laboratory of Sichuan Province, West China Hospital, Sichuan University, Chengdu, 610041 Sichuan People’s Republic of China; 3 Department of Rehabilitation Medicine, The First People’s Hospital of Shuangliu District, Chengdu, People’s Republic of China; 4 Department of Rehabilitation Medicine, First Medical Center of Chinese, PLA General Hospital, 28 Fuxing Road, Beijing, 100853 People’s Republic of China

**Keywords:** Knee osteoarthritis, Power cycling, Neuromuscular exercise, Internet of Things, Telerehabilitation

## Abstract

**Background:**

Osteoarthritis (OA) is a common and highly disabling disease that imposes a heavy burden on individuals and society. Although physical therapy is recommended as an important method to relieve OA symptoms, patients cannot continue treatment after returning home. Research on Internet telerehabilitation for knee osteoarthritis (KOA) can reduce pain and improve patient quality of life, and Internet of Things (IoT)-based telerehabilitation is a new form of delivering rehabilitation. This study will evaluate the effect of telerehabilitation via IoT, as a medium to deliver exercises, on pain and walking in patients with KOA.

**Methods:**

This study is a single-blind randomized controlled trial. We will recruit 42 middle-aged and elderly patients with KOA aged ≥ 50 years and randomly divided into power cycling group, neuromuscular exercise group, and control group, and intervention will last for 12 weeks. Outcome measures will be taken at baseline and 4 weeks, 8 weeks, and 12 weeks post-intervention. The pre- and posttreatment differences in knee pain and physical function between participants undergoing power cycling and neuromuscular training and those in the control group will be determined by each scale. The effectiveness will be assessed by the Western Ontario and McMaster Universities Osteoarthritis Index Score (WOMAC) and an 11-point numerical pain rating scale. Walking function and quality of life will be assessed by the timed up and go and walk test, 6-min walk test, and quality of life health status questionnaires.

**Discussion:**

The findings from this trial will establish the feasibility and effectiveness of IoT-based power cycling and neuromuscular training on elderly patients with KOA in the community. As a result, this trial may help provide experimental evidence for finding a better exercise method suitable for elderly patients with KOA in the community.

**Trail registration:**

Chinese Clinical Trials Registry ChiCTR2200058924. Prospectively registered on 6 May 2022.

**Supplementary Information:**

The online version contains supplementary material available at 10.1186/s13063-022-06946-x.

## Background

Osteoarthritis (OA) refers to a chronic degenerative bone and joint disease caused by a variety of factors, and its clinical symptoms include joint pain, deformity, and motor dysfunction [1]. Globally, 9.6% of men and 18.0% of women aged 60 years and older are living with symptomatic osteoarthritis [2, 3]. OA is widely recognized as a major health problem [4], affecting the quality of life of elderly patients and leading to a huge economic burden [5–7]. It has been reported that nearly 250 million individuals suffered from OA worldwide, and the overall prevalence of primary OA in people over 40 years old in China is as high as 46.3% [5, 8]. OA prone to occur in the knee, hip, and hand joints, of which the knee joints are the most commonly affected site according to previous report [5]. Epidemiological survey showed that the incidence of knee osteoarthritis (KOA) is approximately 16–17%, with an increasing trend year by year. In addition, patients aged among 50 to 70 are more likely to be affected [6, 7]. Exercise therapy, as a core option for KOA treatment, has been recommended by various guidelines [9–11]. Exercise with on-site supervision given by physiotherapists has been proven effective for patients with KOA [5]. However, patients who in rural and remote area, from where the population accounts for a large proportion [8], may have difficulties in getting access to in person guidance when exercising; therefore, to establish a way, prescribing exercises should be incorporated for consideration in the development of a treatment plan that bridges physicians and patients. Telerehabilitation has emerged since 1998 [12]. Telerehabilitation refers to providing patients with continuous rehabilitation services through the use of information and communication technology, with combination with telemedicine [13]. The main contents of telerehabilitation include health education, exercise training, self-management, online follow-up, and regular assessment [13, 14]. Previous study demonstrated the feasibility and safety of telerehabilitation in practical applications [15]. Comparing with in-person forms, telerehabilitation showed no statistic difference in the fields of reducing pain and improving patients’ quality of life [14, 16]. With the popularity of the Internet among the elderly population and the development of modern technology, the platform of telerehabilitation has gradually transitioned from telephone to the Internet and the emerging Internet of Things (IoT) [14, 17]. The Internet of Things refers to a network that is extended on the basis of the Internet, and the user terminal can be extended to anything for information exchange, communication, and more intelligent interactive mode, which is a newly developed form of telerehabilitation. Studies have proven that compared with telephone and DVD digital discs, the guidance of the IoT can improve patients’ self-efficacy [18, 19].

A recent review has put forward a city planning concept, highlighting on constructing a healthy and sustainable city [20]. To achieve this goal, it is indispensable to take medical treatment into consideration, especially for those specific types of chronic non-infectious diseases. For patients with KOA, it takes a lot of time for them to go for doctors or physiotherapists in hospitals on-site, which brings heavy medical and social burdens; therefore, KOA treatment will shift to IoT form, which will be an inevitable trend. Our previous research on the Chinese model of IoT-based KOA rehabilitation showed that IoT rehabilitation can ensure that doctors and therapists keep abreast of the patient’s condition and give appropriate treatment, while also strengthening the communication between doctors and patients [21]. Combining IoT with patient exercise to provide community rehabilitation, giving patients living in remote areas the opportunity to communicate with physicians in real-time, supporting ongoing rehabilitation services for patients with KOA [13, 22], has the potential to increase engagement and outcomes for patients with KOA.

Due to the poor physical function of elderly patients with KOA, low-intensity, slow-moving aerobic exercise should be recommended. Power cycling is a low-load and no-impact form of aerobic exercise that help patients with KOA alleviate pain improve range of motion, muscle strength of lower extremities, and cardiorespiratory endurance [23]. In addition, neuromuscular training, as one of another commonly used clinical exercise for KOA patients, has received increasing attention due to its simple and easy-to-learn exercises. Previous studies have demonstrated that regular power cycling and neuromuscular training can both relieve knee pain and stiffness, improve motor function of lower extremity and patients’ aerobic exercise capacity, and lower extremity strength and quality of life for KOA patients [24–30]. In summary, we have learned about the benefits and convenience of power cycling, neuromuscular training, and telerehabilitation in the rehabilitation of patients with KOA. To better deliver the above exercises, we need to further clarify how the combination of the IoT and specific rehabilitation therapy can help patients with KOA in the community. The IoT has been widely used in various fields of social life, and the medical field is also being studied in depth. Several studies have been conducted, including projects such as “Join2Move,” “PainCOACH,” and “Help My Knees,” to explore the feasibility and effectiveness of Internet-based rehabilitation for patients with KOA [31, 32]. However, to our knowledge, no studies have demonstrated whether power cycling and neuromuscular training via IoT can effectively reduce pain and promote improvements in physical function and quality of life in patients with KOA in the community.

## Methods

### Aim

The primary objective of this study is to compare the effect of IoT-based power cycling and neuromuscular training on the elderly patients with KOA in the community. Whether the intervention of elderly patients with KOA is effective or not provides experimental evidence for finding a better exercise delivery mode for elderly patients with KOA in the community.

### Study design

This study is a 12-week single-blind randomized controlled trial. The trial intervention and data collection will be completed from September 2022 to June 2023. The protocol was designed following the Recommendations for Interventional Trials (SPIRIT) and SPIRIT checklist could be found in Additional file 1 [33]. The trial will be conducted in the community in Chengdu, Sichuan Province. Assessments would perform online.

### Participants and setting

We will recruit 42 participants at West China Hospital, Sichuan University, in Southwest China. Ethics approval was obtained from Ethics Committee on Biomedical Research, West China Hospital of Sichuan University, and prospectively registered on the Chinese Clinical Trials Registry (ChiCTR2200058924) registered 6 May 2022.

### Inclusion criteria

Participants are potentially eligible if they (i) have knee pain and could provide with previous diagnosis certificate from a regular medical institution or are diagnosed with KOA according to the Guidelines for Diagnosis and Treatment of Osteoarthritis in China (2021 Edition) (see Table 1) [8]; (ii) have a I–III Kellgren-Lawrence grade [34]; (iii) are able to use their smartphones to send messages and make video calls using WeChat; and (iv) can communicate in Mandarin and understand Chinese characters.

**Table 1:** Inclusion and exclusion criteria

Inclusion criteria
1. Has knee pain and is diagnosed with KOA (provide a relevant diagnosis certificate from a regular medical institution)
2. Accord with following (1) and two criteria from (2) or (3) or (4) or (5):
(1) Recurrent knee pain in the last month
(2) Narrowed joint space, subchondral cyst formation and bone sclerosis, or osteophytosis around joint margin on the radiographs in standing or load position in X-ray
(3) Aged 50 years and over
(4) Stiffness ≤ 30 min in the morning
(5) Palpable bone crepitation (fremitus) on movement of joint
3. Kellgren-Lawrence grade I–III of KOA
4. Be able to use smartphones with an Internet connection and send text messages
5. Be able to communicate in Mandarin and understand Chinese characters
**Exclusion criteria**
1. Diagnosed with rheumatoid arthritis, severe osteoporosis, or other pathological conditions of the knee joint
2. Knee replacement or other knee surgery
3. Receive rehabilitation treatment for KOA in hospitals, clinics, or rehabilitation institutions or participate in other projects related to KOA rehabilitation
4. Uncontrolled hypertension and diabetes (such as fasting blood glucose ≥ 16.7 mmol/L) or repeated episodes of hypoglycemia (blood glucose ≤ 3.9 mmol/L) or other diseases that restrict exercise (such as stroke, heart failure, and severe anemia)
5. Patients with a history of mental disorders
6. Being informed by a doctor that there are other circumstances in which it is inappropriate to exercise without supervision
7. Unable to sign informed consent
8. Unable to communicate with others in normal Chinese
9. Cannot use smartphone or WeChat to send messages and have video calls

### Exclusion criteria

Participants who meet any of the following criteria will not be able to participate in this study if they (i) have rheumatoid arthritis, severe osteoporosis, or other pathological conditions at the knee; (ii) are ready for knee arthroplasty or other knee surgery; (iii) receive KOA rehabilitation treatment or participating in other programs related to KOA in hospitals, clinics, or rehabilitation institutions; (iv) undergo uncontrolled hypertension and diabetes (such as fasting blood glucose ≥ 16.7 mmol/L), repeated episodes of hypoglycemia (blood glucose ≤ 3.9 mmol/L), or other diseases that restrict exercise (such as stroke, heart failure), severe anemia, etc.; (v) are with a history of mental disorders; and (vi) are informed that there are other circumstances that are not suitable for exercises without supervision; (vii) are unable to sign an informed consent.

### Randomization and allocation

After the participants completed the baseline measurements, they were divided into power cycling group, neuromuscular training group, and control group according to the random allocation principle using a computer-generated random allocation table at a ratio of 1:1:1. Then, according to the random number, the corresponding test envelope is obtained, which includes the form for recording participants' information, the intervention plan, and the plan for emergent event to ensure that each participant was only aware of their own experimental interventions.

### Blinding

Due to the characteristic of this trial, participants and physiotherapists will not be blinded to group allocation and will be aware of the alternative treatment options. The study hypotheses will be blinded to participants. The researcher conducting the data collection and analysis will be blinded to treatment allocation.

### Participant timeline

**Fig. 1:**
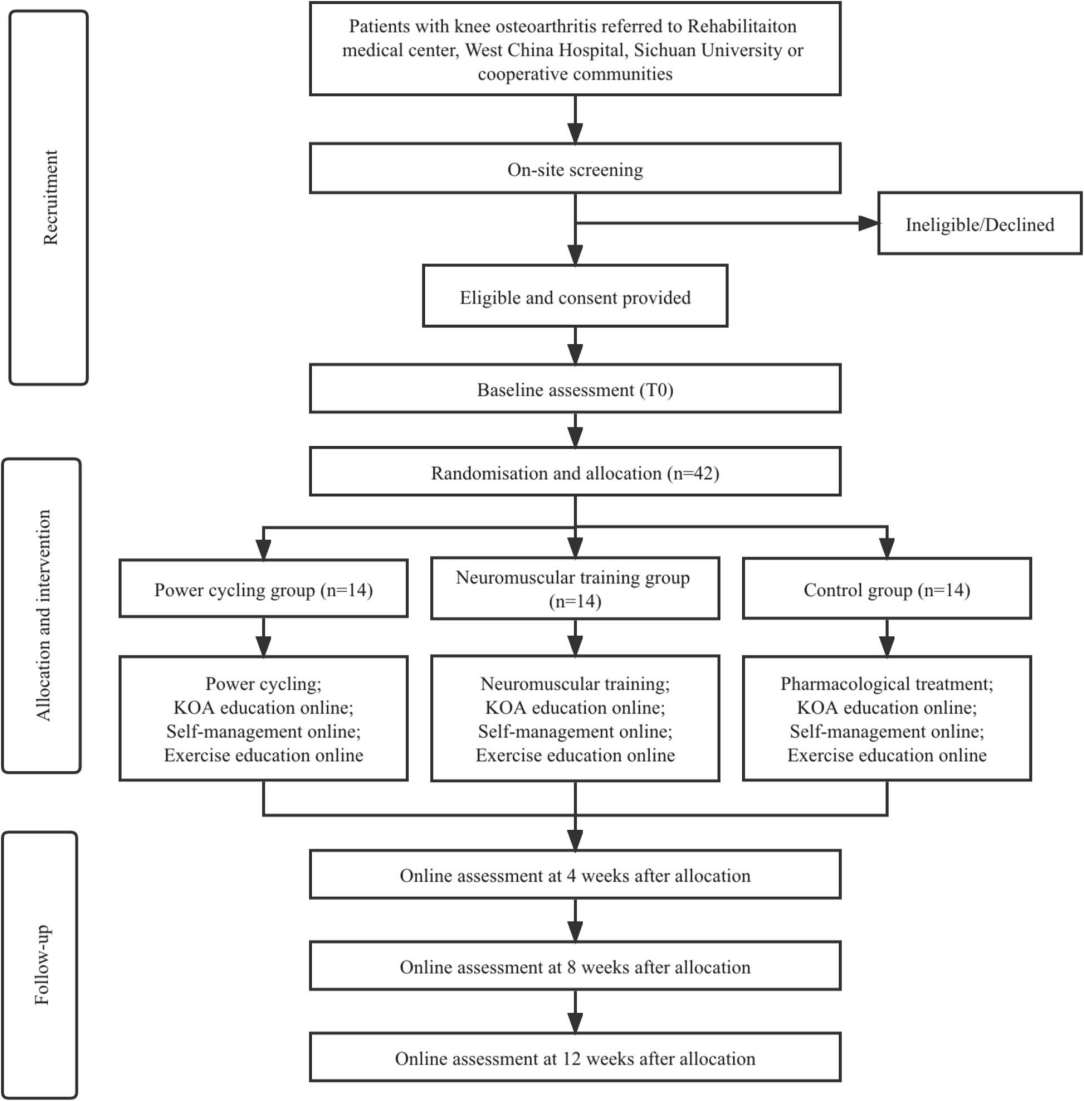
Flow-chart of the study

Table 2 shows the assessments at each time point following the SPIRIT statement [33]. Figure 1 demonstrates the flow chart of this trial.

**Table 2:**
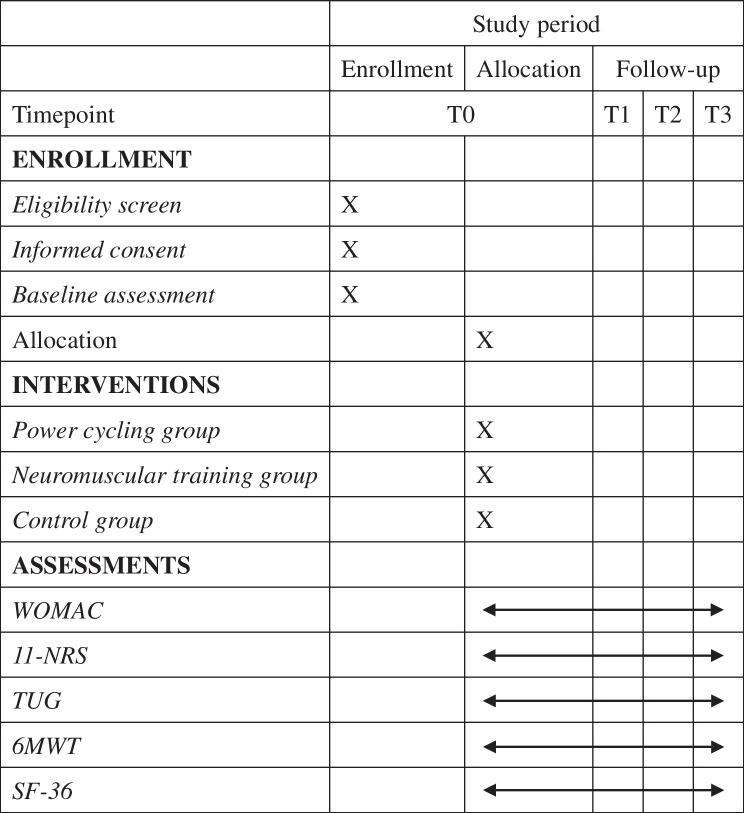
Study assessments at specific time points

### Media for intervention prescription

To realize the connection between Internet and things, we built an applet, called Huaxi Rehabilitation Cloud applet, within the WeChat mini programs platform, by which the participants could receive the prescription of the corresponding interventions. To make it more convenient for usage, we invited public and therapists to involve in the design process of this applet and asked for any suggestions during using the applet in daily routine. After several internal discussions, we optimized the final version (Additional file 2) of the applet and applied in our trial.

### Power cycling group

Participants in this group will conduct three 30-min cycling sessions per week and twelve weeks in total. Participants will perform exercise with power cycling which are set in community before our study launched. Each time participant warms up before exercises and cool down for 5 min after that. The exercise intensity, measured by the rating of perceived exertion (RPE), scores 12–14 points during cycling, that is, with self-efficacy of feeling a little hard [35]. A physiotherapist will supervise participants through a video call weekly, and new exercise prescription will be delivered when participants feel easy or too hard to perform cycling at the pre-set intensity. Doctors and physiotherapists will conduct telerehabilitation assessments, after which they will send exercise prescriptions through applet according to the assessment results. Then, participants scan the QR code typed on the power cycling through the applet to start exercise. If participants have any discomfort during exercise, they can click “End Early” on mobile phone to stop, and the physiotherapist will automatically receive a feedback, and they will decide whether it is necessary to make a new assessment. Besides, the weekly video call session and progressive exercise prescribed by physiotherapist could be considered as a method to monitor potential dropout issue and prevent the loss of follow-up and reduce the number of subjects who violate the allocation plan.

### Neuromuscular training group

Participants allocated in this group will conduct exercises at home three times per week [36]. Details of neuromuscular training exercises could be found in Table 3 [28]. Generally, it comprises weight-bearing training and up-down stepping exercises. After assessments, doctors and physiotherapists will send training videos to participants and have a video call to ensure the exercises are performed correctly. Family members will accompany the participants during each exercise session (including video call with physiotherapists) to ensure safety and to provide any support. Physiotherapists will prescribe planned exercises according to the feedback received from participants weekly. If pain worsen and could not relieve within 24 h after conducting exercises advanced, participants will be asked to continue the former exercises.

**Table 3:** Details of neuromuscular training exercises

	**Exercise dose:** 10 sets/session, 3 sessions/time (with a 30–60-s rest between each session), 3 times/week, 30 min/time, 12 weeks in total	
	**Exercise 1**	**Exercise 2**
Weeks 1–2	**Starting position:** standing, feet shoulder-width apart, and the affected side is holding a fixed table **During exercise:** Unaffected lower extremity keeps straight and slides forward and backward on the flat ground, while the affected side, as a support, flexes at the same time. When the unaffected side slides back, the weight-bearing side extends gradually from the flexion position. Participants are required to keep the alignment of the hip, knee, and ankle alignment and place the center of gravity on the affected side	**Step up:** affected side goes up first, with the center of gravity placed on the lower extremity on the affected side. During the flexion-straight process, the affected side is required to perform as slowly as possible, be kept stable, and avoid shake **Step down:** Unaffected side goes down first. Participants complete the action of descending the steps by controlling the affected side to gradually flex from extension. Being slow, being stable, and avoiding shake like up step exercise are also required
Weeks 3–4	On the basis of weeks 1–2, additionally provide a laterally exerted resistance on the affected joint by using elastic band (Thera-Band, yellow). Participants are required to maintain correct alignment of the lower extremity under resistance (hip, knee, and ankle are on the same sagittal plane)	On the basis of weeks 1–2, participants are asked to hold dumbbells of 1 kg in each hand
Weeks 5–6	The same as weeks 3–4, except adding resistance (red elastic band)	The same as weeks 3–4, except adding resistance (2 kg in each hand)
Weeks 7–8	The same as weeks 3–4, except adding resistance (green elastic band)	The same as weeks 3–4, except adding resistance (3 kg in each hand)
Weeks 9–12	The same as weeks 9–12	The same as weeks 9–12

Reference: [28]

### Control group

According to the recommendation by Chinese guideline for diagnosis and treatment of osteoarthritis (2021 edition) and Osteoarthritis Research Society International (OARSI), participants are administrated with glucosamine hydrochloride capsules (pharmaceutical product registration certificate number HC20140008 and produced by Hong Kong Aomei Pharmaceutical Factory), with a dose of 750 mg and 1.5 g/day, to relieve symptoms of KOA [8, 11].

### Common interventions

In addition to the specific and individual interventions for three groups mentioned above, we also set three aspects as common rehabilitation interventions and participants in each group will receive the following information:

- KOA education
  Through applet, information detailing the knowledge of KOA and clinical guidelines were sent three times per week by doctors and physiotherapists. At the same time, participants can also communicate with healthcare providers through Huaxi Rehabilitation Cloud applet to inquire any health conditions about KOA.
- Self-management
  Information comprise weight management and emotional management.
  (a) Weight management: obesity is one of the risk factors of KOA, so here, we emphasize weight control [1]. Participants need to upload their height and weight to the applets every week, and the system will automatically generate a body mass index (BMI). When BMI > 24 kg/m^2^, the doctor will remind the participant to control their weight and send information about weight management and healthy eating. (b) Emotional management: patients with chronic pain and activity limitation are at a high risk of depression [37], and in this study, physical therapists push brochures to participants and communicate and guide them. Ensure adequate sleep (7–8 h a day), chat with friends every day, take a deep breath when troubled, listen to soothing light music when irritable, etc.
- Exercise education

Participants should do at least 150 min of moderate-intensity (70% of maximum heart rate) aerobic activity per week, such as brisk walking, swimming (freestyle is best), and cycling. Excessive exercise should be avoided, and knee pads and other auxiliary devices should be used to support and protect the diseased joints. You can try team sports under the guidance of a special person, such as group hiking and group gymnastics., in mutual supervision and encouragement, to increase the fun of sports.

### Outcome assessments

Outcomes will be measured at baseline (T0) and at 4 (T1), 8 (T2), and 12 (T3) weeks after the allocation. All participants will be assessed by researchers blinded to group tasks.

### Primary outcome measures

(I) The Western Ontario and McMaster Universities Index (WOMAC) score will be used to assess knee pain, stiffness and physical function. The WOMAC is a self-assessed health status scale for KOA patients consisting of 24 items in three subscales of pain (5 items), stiffness (2 items), and physical function (17 items). All items are scored from 0 (asymptomatic) to 4 (very severe), and the total score ranges from 0 to 96, with higher scores indicating more severe symptoms [38, 39]. The test–retest reliability of the scale was 0.83, and the validity was 0.77 [40]. (ii) The 11-point numerical pain rating scale (11-NRS) was used to assess knee pain 48 h before the test. The 11-NRS is a standard tool for the study of chronic pain. A score of 0 indicates no pain, and 10 indicates severe pain [41].

### Secondary outcome measures

(I) Lower limb strength and balance determined by timed up and go (TUG) test. Record the time the following process takes: stand up from a standard seat (seat height is approximately 46 cm, armrest height is 65 cm), walk 3 m away then turn around, walk back to the chair, and sit down [42–44]. (ii) 6-min walk test (6MWT): In an outdoor closed corridor (30 m), participants were allowed to walk back and forth. The timer is set to 6 min, and total walking distance is measured. Participants are allowed to slow down and stop to rest if necessary but are encouraged to continue walking [42, 45]. (iii) Short Form 36 Item Health Survey (SF-36) includes 8 dimensions: physical function, physiological function, physical pain, general health, vitality, social function, emotional function, and mental health, with a total of 36 questions. The total score ranges from 0 to 100. A higher total score indicates a higher quality of life [46, 47].

### Safety measure

Since the majority of the interventions are physical exercises, there is no anticipated harm and compensation for trial participation. Participants will be monitored for any adverse events during the intervention, which will be collected and recorded on the case report form (CRF). All adverse events and abnormal laboratory parameters will be followed up until normal, abnormal clinical significance or return to the pretreatment state.

### Sample size

The calculation is carried out according to the unified standard of clinical trial reporting and the calculation requirements of the sample size of noninferiority experiments [41, 48]. Based on the previous research results of our research team and comprehensive consideration of previous experience [28], the reduction in pain score in the WOMAC scale was used to assess the improvement of KOA symptoms. The average pain scores in patients with KOA were 1.65, 1.7, and 0 respectively [36, 49]. We set a to 0.05 and test power 1-β to 90%. Calculation via G*Power 3.1 software. A total of 33 participants is required for the calculation. With allowance for a dropout rate of 20%, each group requires 14 enrolled participants.

### Statistical analysis

Statistical analysis is performed using the SPSS software (25.0). Significance level will be set at 0.05 for all statistical analysis. Intention-to-treat principle will be followed. Baseline comparability between groups of categorical variable data was determined using the chi-square test or other suitable nonparametric tests. Quantitative data will be expressed as the mean (SD), if it follows normal distribution and homogeneity of variance; otherwise, median (IQR) is given. For statistical inference, if the data obeyed a normal distribution and homogeneity of variance and satisfied spherical symmetry, variance analysis of repeated measurements was used; otherwise, the Kruskal–Wallis *H* rank sum test will be conducted. For data that recorded from drop-out participants, maximum likelihood estimation will be used to impute missing data, including any lacks for primary and secondary outcomes. The data collected are kept by people not involved in the trial intervention and by the online platform. The datasets analyzed during the current study and statistical code are available from the corresponding author on reasonable request, as it is the full protocol.

### Data collection and monitoring

An independent Data Safety Monitoring Committee will be assembled to assess the safety and validity of the execution of the trial as well as to ensure the best interests of participants will be observed at all times. The committee will be comprised by a chairman, one independent statisticians and two physiotherapists, who will be responsible for (1) monitoring participants’ safety at all times during the trial; (2) noting any reported adverse events, making medical decisions and providing essential management to control the situation; and (3) reviewing participant recruitment and withdrawal and mark down the reasons. Once 10, 50, and 80% of the sample size is reached, a data quality audit will be performed during the trial being conducted. Further, data will be stored in encrypted spreadsheets on secured servers hosted by the West China Hospital of Sichuan University, in which any potential risk of omissions and errors will be regularly scrutinized, and then exported to a statistical software for analysis by a statistician blinded to group allocation. All data collected in this trial will be restricted to the principal investigator and specific members of the research team using the backend of the database or servers. A monitoring committee (The Good Clinical Practice Unit at West China Hospitals) will follow and oversee this trial. The monitor will visit the trial site before trial commencement and, after that, have a visit of the trial site twice per month. The monitor who is in charge will check trial procedures, including the completion rate and quality of prescribed exercises, safety assessments, and data recording, and complete the verification procedures of raw data and participant confidentiality. For the enrollment and follow-up of participants, a trial steering committee will manage the trial process and check day-to-day. Any suspension of participation caused by low adherence or voluntary quit by the influence of the COVID-19 pandemic will be recorded.

### Ethics approval and dissemination

The trial will be conducted in accordance with the Declaration of Helsinki. Ethics approval was granted by the Clinical Trial and Biomedical Ethics Committee of the West China Hospital, Sichuan University (ethics reference: 2021 (1696)). The study was registered on the Chinese Registry website (ChiCTR2200058924). Informed consent will be provided to all participants and signed prior to the trial. All study-related information of subjects will be protected during the trial. On the consent form, participants will be asked if they agree to collect and use of their data should they choose to withdraw from the trial, and this trial does not involve any biological specimens. The consent form is available, on request, from the corresponding author.

The results of this study will be published at local, national, or international rehabilitation conferences and submitted as manuscripts to peer-reviewed journals. The main findings of the study will also be shared with all participants and disseminated to researchers, health service providers, health care professionals, and the public via the courses.

## Discussion

This study aims to investigate the effect of Internet-based power cycling or neuromuscular training on pain and walking function in KOA patients. Unlike traditional research, we enroll eligible participants through outpatient clinic, communities, and the Internet. Our rehabilitation setting will focus primarily on the community. After allocation, we will provide them with health education and a personalized exercise training plan. The entire process will use online video connections and platforms to implement interventions. The exercise process requires community administrators and family members to supervise and provide any support. During telerehabilitation, participants will have opportunities to communicate with their peers in a community setting, thereby promoting willingness and motivation to participate through mutual encouragement [50]. Besides, performing neuromuscular training at home saves patients’ time commuting to hospitals. During this process, community staffs can participate in the professional training of constructing community KOA network rehabilitation to improve the whole-cycle rehabilitation treatment for patients with KOA, which plays an active role in improving community rehabilitation and reducing the burden on individuals and society. The findings of this study may help develop an evidence-based online exercise program to manage symptoms and function in patients with KOA in the community. However, the proposed study also has some potential limitations. First of all, during the implementation of the research plan, participants may not be recruited on time due to the impact of the epidemic, we will expand the online recruitment channels for this. Secondly, there is no evidence-based quantitative tool to record the frequency and details of family members’ companionship during exercise; we will make a patient exercise card-level adverse event table and ask family members and community administrators to make detailed records of the frequency and details of exercise companionship and adverse events.

## Trial status

Recruitment will be started on 1 September 2022, and it is estimated to have a total duration of 10 months. This protocol is the version number 5 dated 2 June 2022.

## Supplementary Information

**Additional file 1: SPIRIT checklist.**

**Additional file 2: Huaxi Rehabilitation Cloud applet construction.**

## Data Availability

Not applicable.

## Abbreviations

OA: Osteoarthritis
KOA: Knee osteoarthritis
IoT: Internet of Things
WOMAC: Osteoarthritis Index Scores from Western Ontario and McMaster Universities
11-NRS: 11-Point numerical pain rating scale
TUG: Timed up and go
6MWT: 6-Min walking test
SF-36: The MOS item short from health survey
OARSI: Osteoarthritis Research Society International
SD: Standard deviation
IQR: Interquartile range
ANOVA: Analysis of variance
QR: Quick response
