# Supplementary material for: Effects of Internet of Things-based power cycling and neuromuscular training on pain and walking ability in elderly patients with KOA: protocol for a randomized controlled trial
Source: Trials. 2022 Dec 13;23:1009. doi: 10.1186/s13063-022-06946-x (PMC9745721; doi:10.1186/s13063-022-06946-x)
Supplement: Supplementary file 2 — Additional file 2: Huaxi Rehabilitation Cloud applet construction. [file 13063_2022_6946_MOESM2_ESM.docx]

**Huaxi Rehabilitation Cloud applet**

**A. Access to the applet**

**
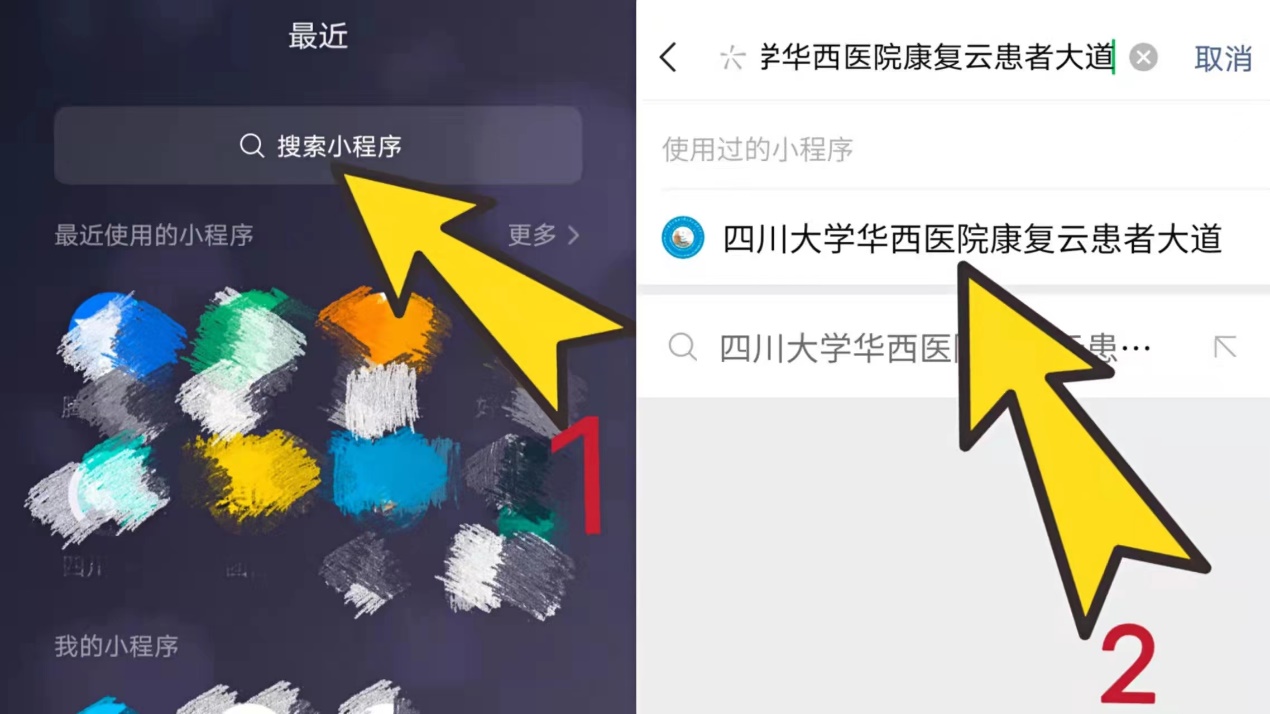
**

**B. Features of the tool and how to bind with doctors**

**
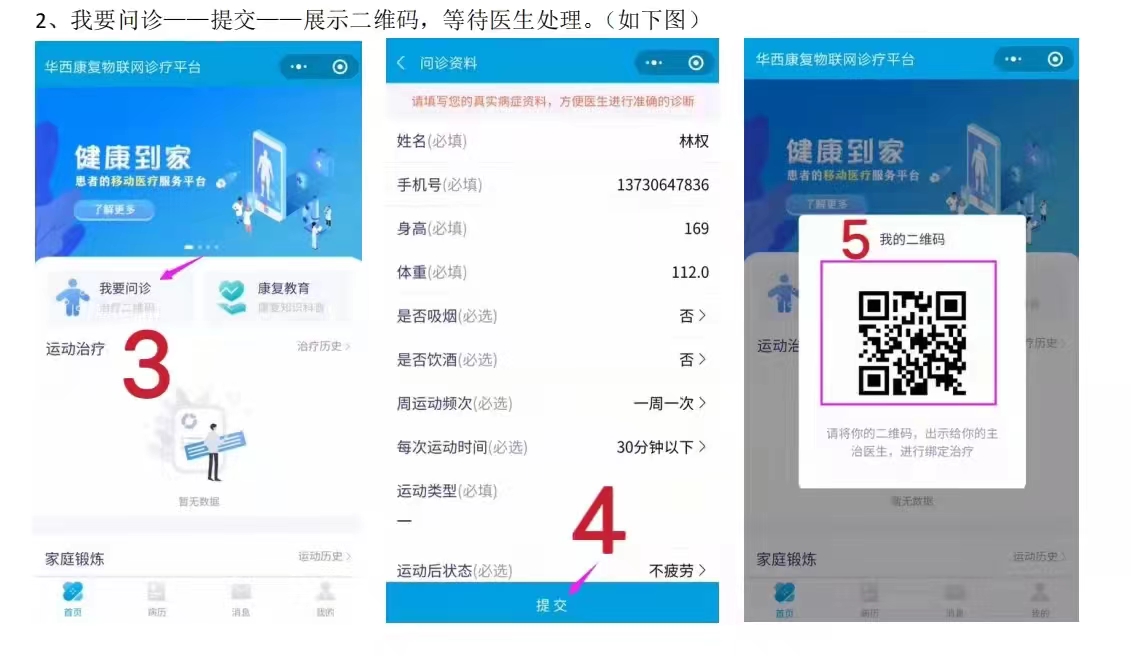
**

**C. Doctors prescribes exercises according to assessment results and participants start cycling or neuromuscular training**

**
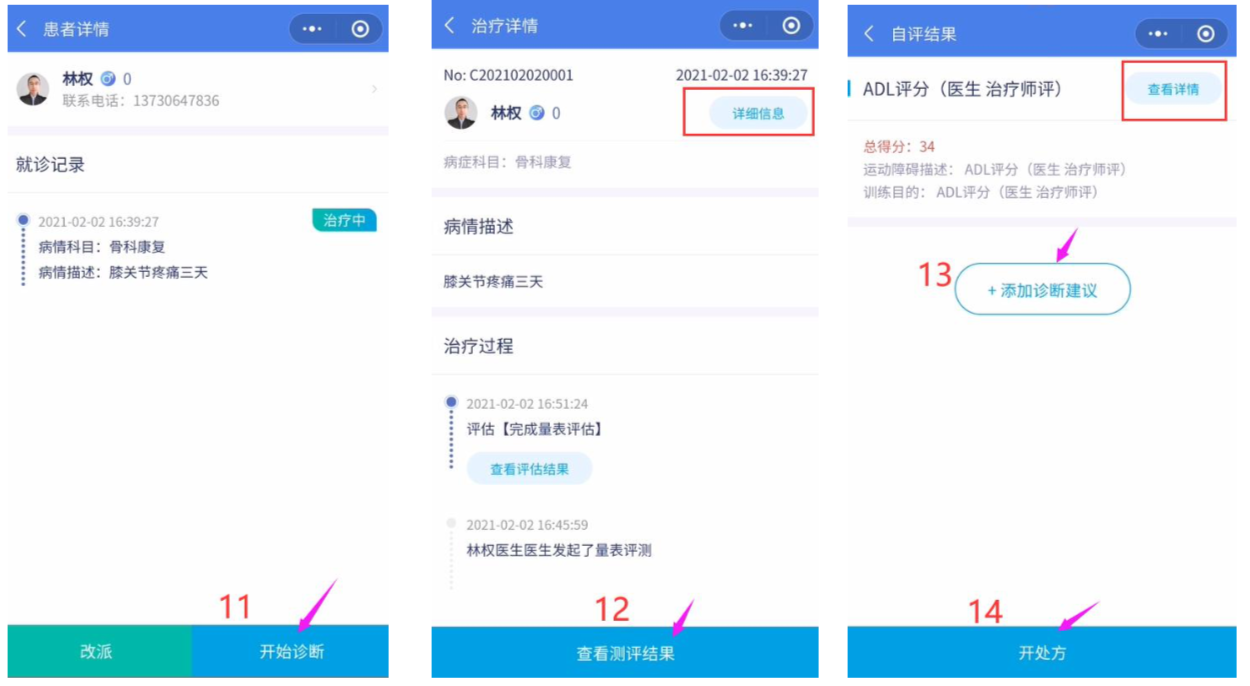
**

**
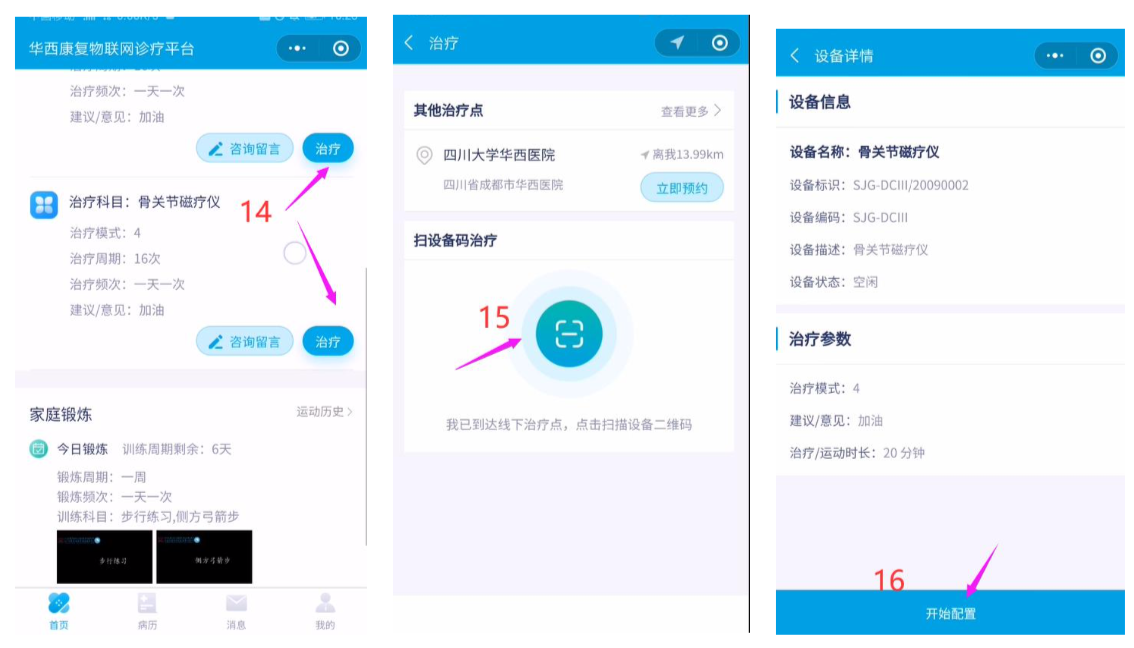
**

**
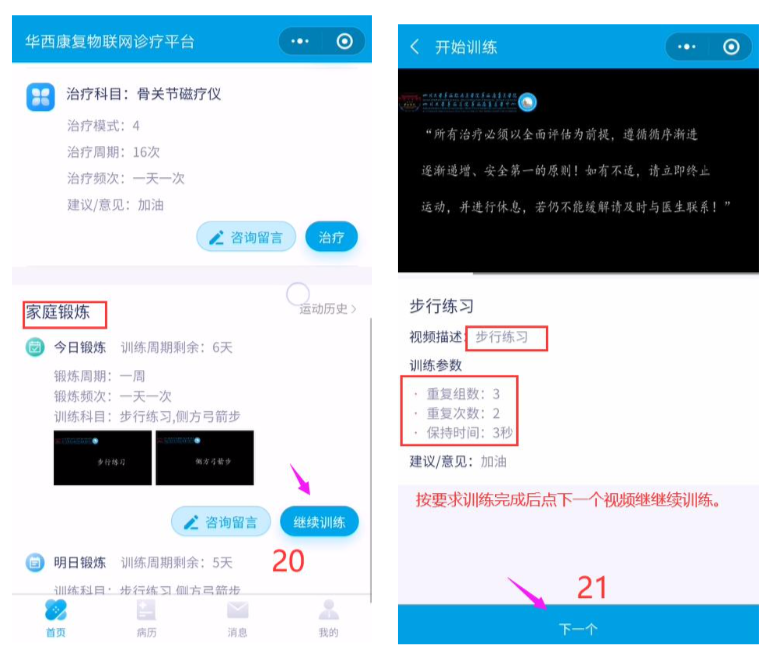
**

**The source of the image:** Five images of the applet were screenshots from the software, and we have arranged the images according to the actual steps participants and physicians follow.

**Permissions to use this image:** The images were screenshots from the software, which was designed and developed by our institution and the computer software copyright registration certificate (original document and its English-translated version) is shown below.


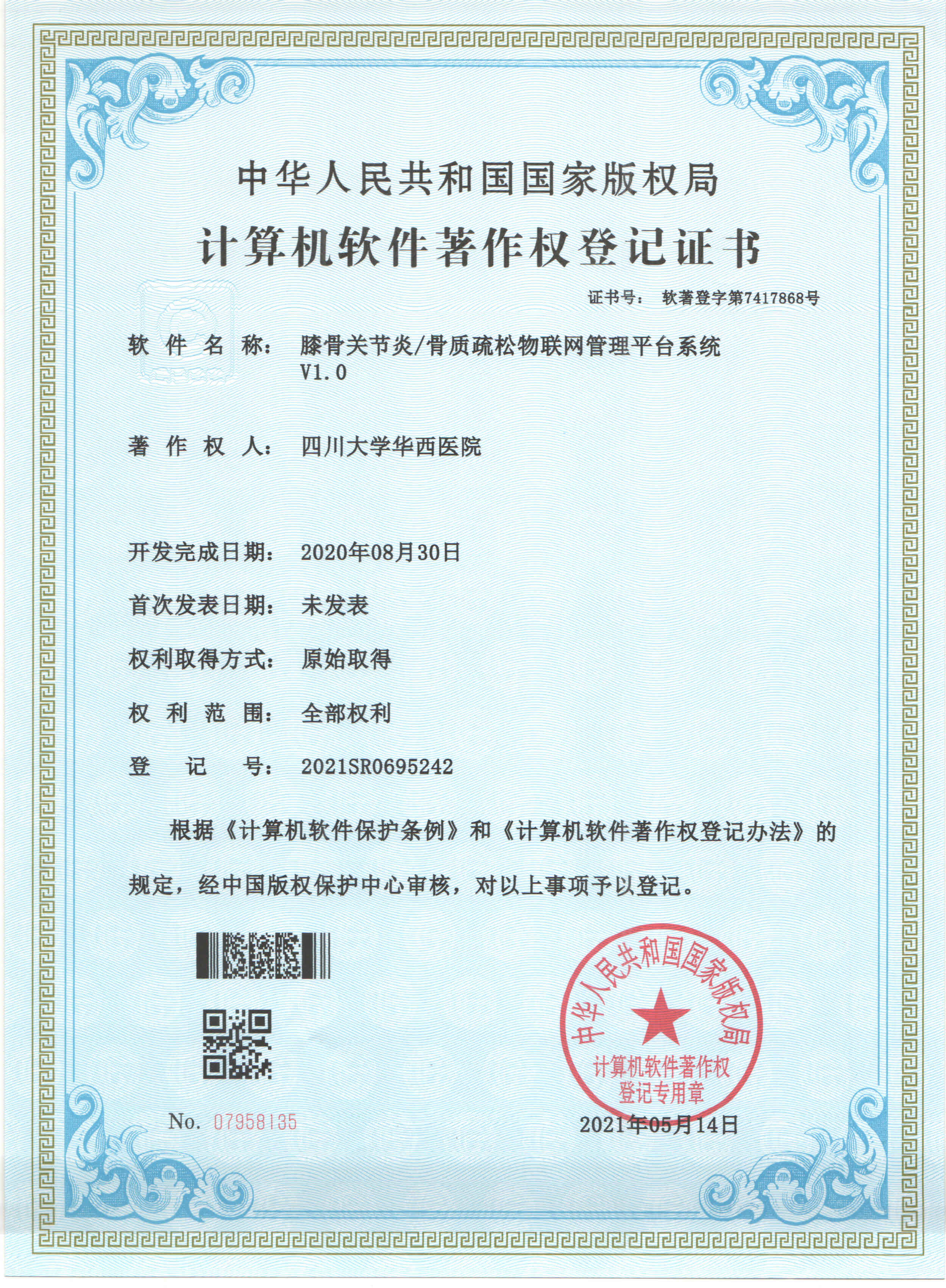


National Copyright Administration of the People's Republic of China

Certificate of copyright Registration of computer Software

Certificate number: soft print No. 7417868

Software name: knee osteoarthritis / osteoporosis Internet of things management platform system V1. 0

Copyright owner: West China Hospital of Sichuan University

Development completion date: 30 August 2020

Date of first publication: not published

Mode of acquisition of rights: original acquisition

Scope of rights: all rights

Registration number: 2021SR0695242

In accordance with the regulations on the Protection of computer Software and the measures for the Registration of copyright in computer Software, it is stipulated that the above matters shall be registered after examination and approval by the China copyright Protection Center.

No. 07958135

National Copyright Administration of the People's Republic of China

Computer software copyright Special seal for registration
